# Supplementary material for: Identification of Bacterial Drug-Resistant Cells by the Convolutional Neural Network in Transmission Electron Microscope Images
Source: Front Microbiol. 2022 Mar 15;13:839718. doi: 10.3389/fmicb.2022.839718 (PMC8965347; doi:10.3389/fmicb.2022.839718)
Supplement: Supplementary file 1 [file Data_Sheet_1.PDF]

SUPPLEMENTARY INFORMATION

Identification of bacterial drug-resistant cells by the convolutional neural network in transmission electron microscope images

Mitsuko Hayashi-Nishino, Kota Aoki, Akihiro Kishimoto, Yuna Takeuchi, Aiko Fukushima, Kazushi Uchida, Tomio Echigo, Yasushi Yagi, Mika Hirose, Kenji Iwasaki, Eitaro Shin'ya, Takashi Washio, Chikara Furusawa, and Kunihiko Nishino

Supplementary Materials

Fig. S1. Sample preparation and data acquisition procedures.

Fig. S2. TEM images of bacterial specimens used for the study.

Fig. S3. Preprocessing of TEM images for cell region extraction.

Fig. S4. Additional Grad-CAM results depicting discriminative parts of cells.

Table S1. Number of patches used for the study.

Table S2. Gene groups correlating highly with image features.

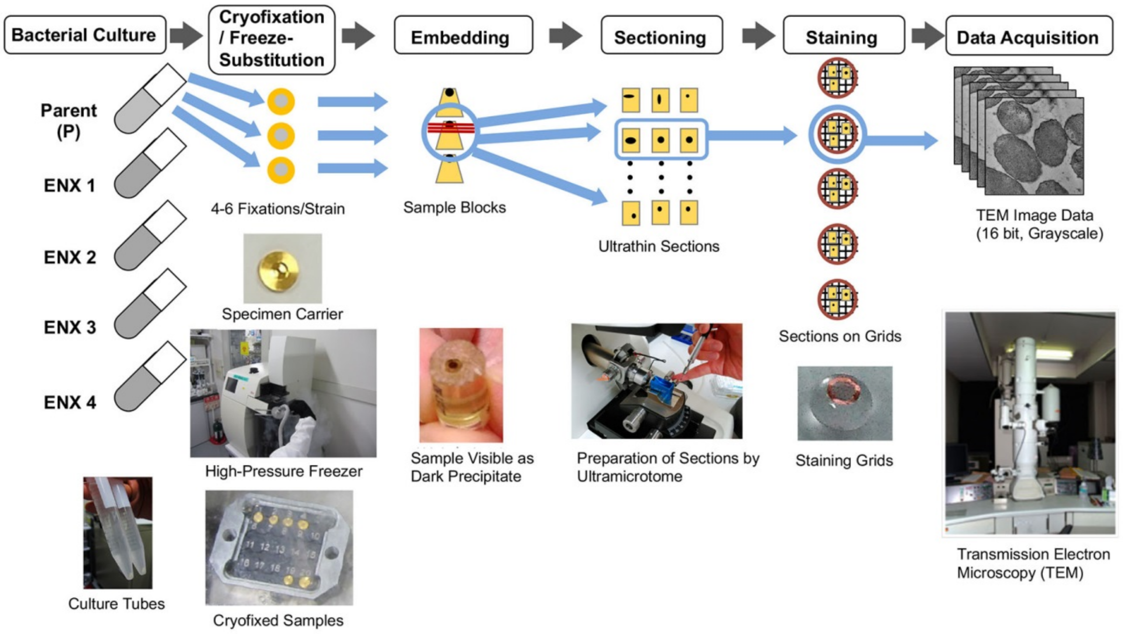

Fig. S1. Sample preparation and data acquisition procedures.

The bacterial cell cultures indicated were centrifuged, transferred to a flat specimen carrier, and cryofixed. Cryofixation was conducted several times for each culture. Freeze-substituted specimens were embedded in epoxy resin to make the specimen blocks. Ultra-thin sections were cut from each specimen block and collected on grids. The grids were counterstained and observed under a TEM. Images of bacterial cells were taken and used for analysis.

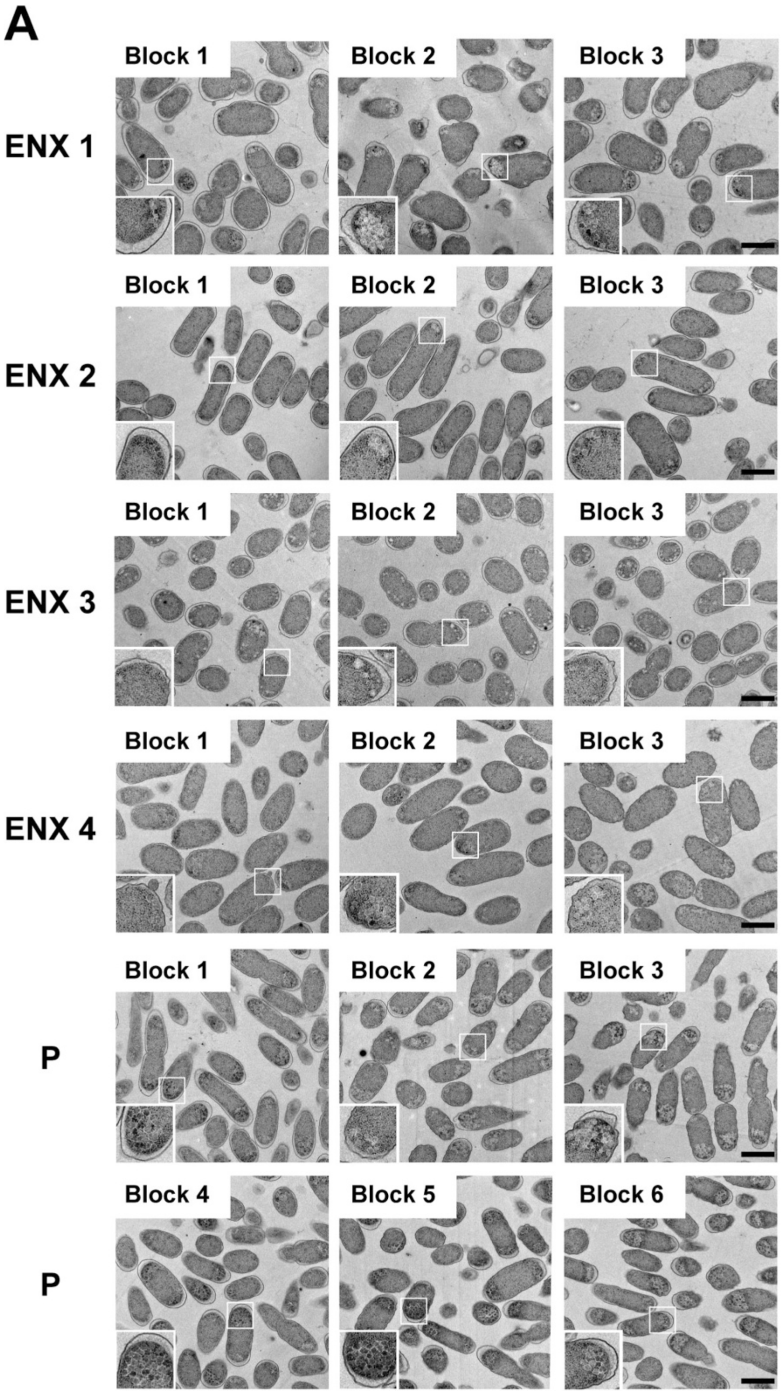
A
ENX 1
ENX 2
ENX 3
ENX 4
P
P
Block 1
Block 2
Block 3
Block 4
Block 5
Block 6

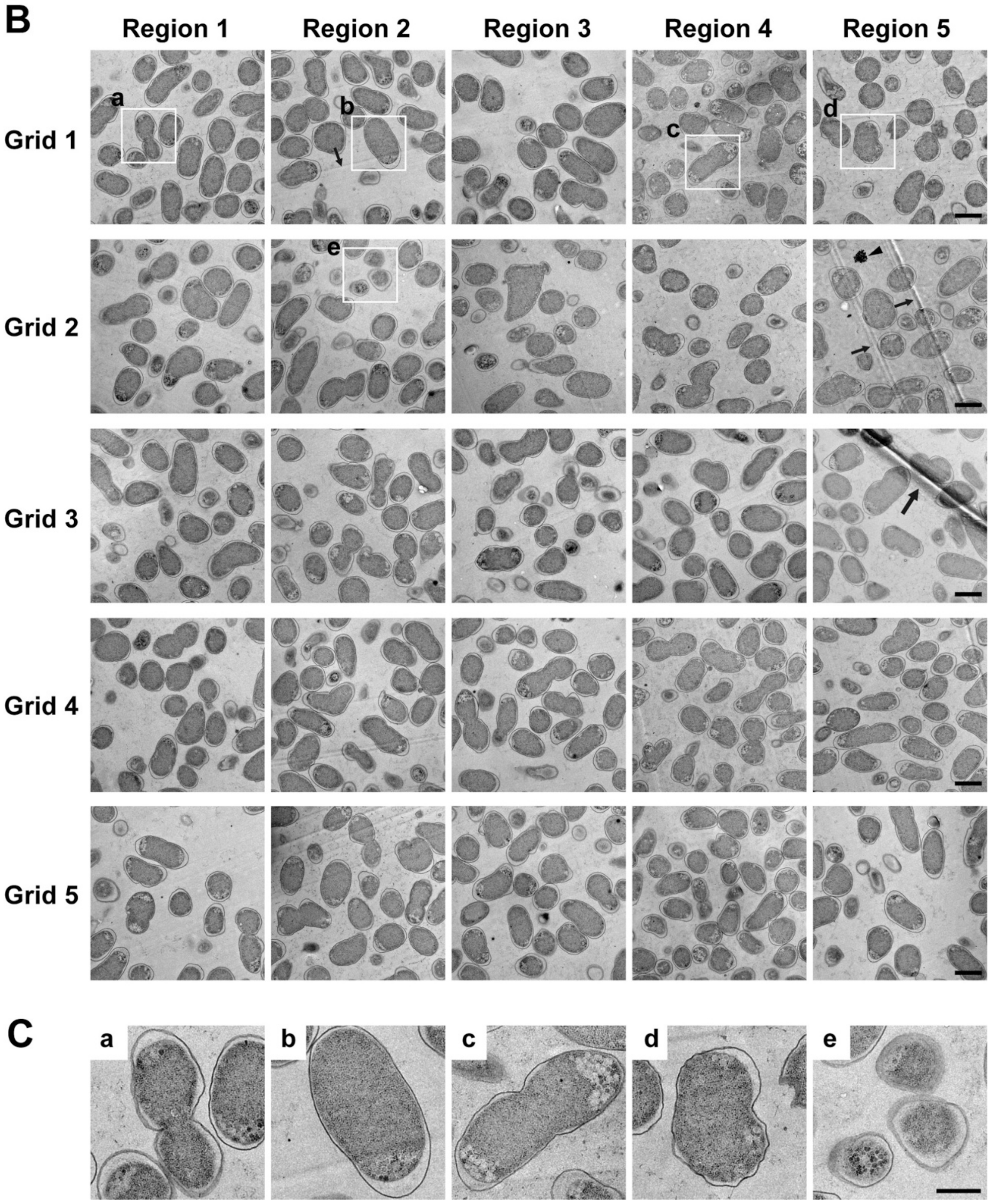

Fig. S2. TEM images of bacterial specimens used for the study.

(A) Representative TEM images of 80-nm-thick sections prepared from individual bacterial specimen blocks used for the study are shown. Insets show the magnification

of the boxed regions in each micrograph, indicating variations in subcellular appearance, such as the smoothness of membranes, periplasmic space, and electron density of granules, among the specimen blocks. (B) Examples of TEM images of ENX 1 (Block 1) are shown. These were obtained from five different arbitrary regions in the sections on five different grids. (C) Enlarged views of the boxed regions in (B) show examples of large variations in the appearance and shape of cells. (a–d) Vertical cross sections of a dividing cell, single cell, a lower contrast cell with less stained granules, and a cell with irregular shape and rattling outer membrane, respectively. (e) Horizontal cross section of three neighboring cells. Examples of knife marks, wrinkle of a section, and noncellular contamination are indicated by arrows, a large arrow, and an arrowhead, respectively. Scale bars in A and B, 1 μm; in C, 500 nm.

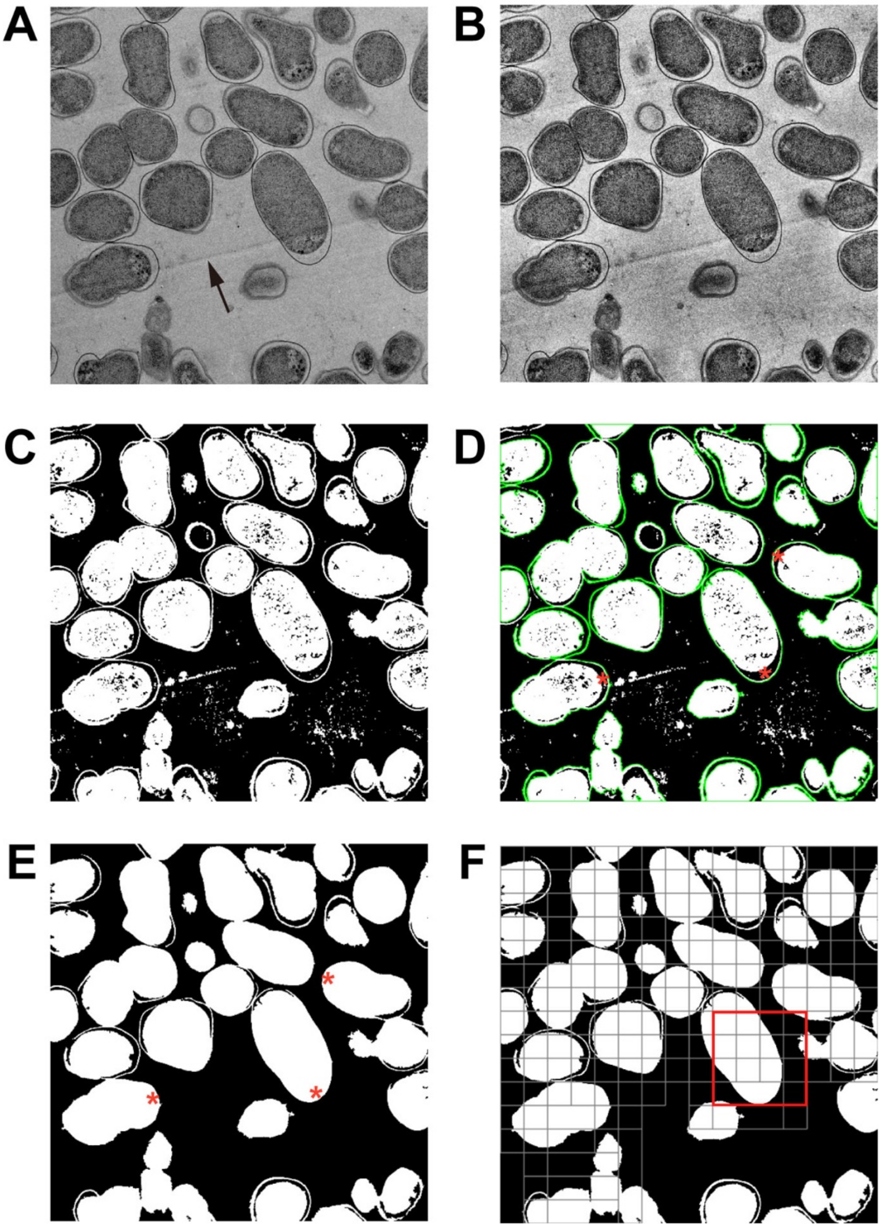

Fig. S3. Preprocessing of TEM images for cell region extraction.

(A) A raw TEM image of ENX 1 (the same image from Fig. S2B, Grid 1, Region 2) with uneven intensity levels (after standardization and conversion into 8 bit). Note that the intensity is brighter at upper right of the image. A knife mark is indicated by an arrow. (B) The TEM image after enhancement of the local contrast by CLAHE. (C) A

mask image obtained by the Otsu method. (D) Most external contours used to fill holes (i.e., enlarged periplasm, indicated by red asterisks) in cell regions. (E) A mask image refined through hole filling and removal of small areas including a knife mark. (F) Extraction of patches from the TEM images. An example of a 512 × 512 patch containing a portion of cells is indicated by the red square.

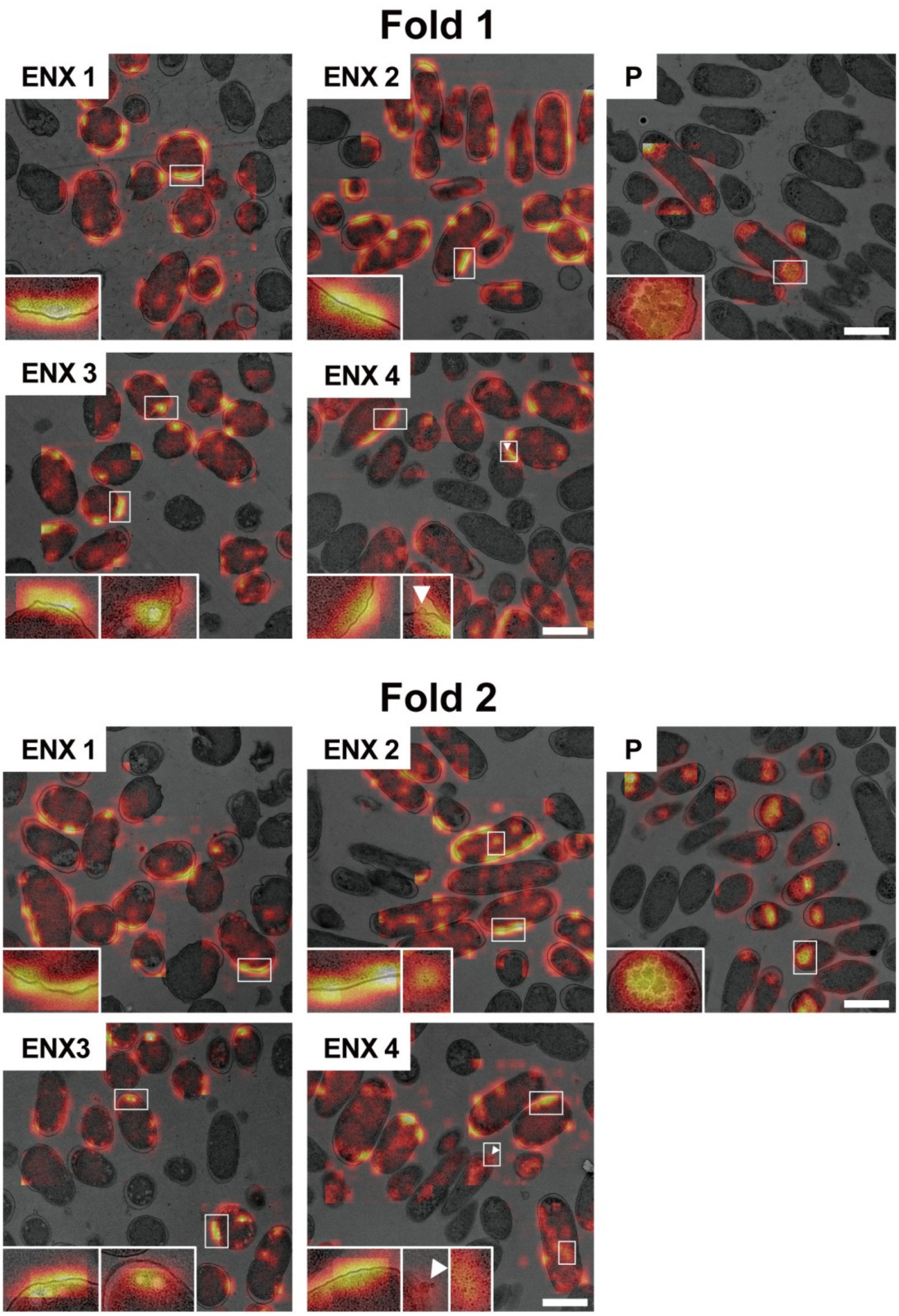

Fig. S4. Additional Grad-CAM results depicting discriminative parts of cells.

Besides the Grad-CAM results demonstrated in Figure 6, similar results obtained from

the other two folds are shown. Insets depict enlarged views of the boxed regions in each image. White arrowheads indicate bleb-like structures. Scale bars, 1 μm.

Table S1. Number of patches used for the study[a].

Fold		Training	Validation	Test
1	Positive	285,478	73,852	236,474
	Negative	231,032	69,444	128,165
2	Positive	328,161	85,702	181,941
	Negative	201,531	69,922	157,188
3	Positive	331,769	86,646	177,389
	Negative	222,399	62,954	143,288

[a]The number of patches extracted from the TEM images and used for training, validation, and test sets, are shown. “Positive” denotes the ENX-resistant strains and “Negative” denotes the parental strain.

Table S2. Gene groups correlating highly with image features[a].

Fold 1				Fold 2				Fold 3		
Gene	Frequency	Mean Value		Gene	Frequency	Mean Value		Gene	Frequency	Mean Value
lpp	6	0.999678		*lpp*	23	0.999633		*lpp*	68	0.99962
ybgJ	5	0.999491		*phoP*	16	0.999538		*phoP*	12	0.99951
asnC	4	0.999403		*pspB*	11	0.999549		*appB*	4	0.99979
aroH	3	0.999182		*nagE*	5	0.999376		*pdxK*	2	0.99938
yedQ	3	0.999350		*rrsH*	3	0.999187		*yacC*	1	0.99985
yheO	3	0.999572		*appB*	3	0.999457		*hlpA*	1	0.99956
carA	2	0.999085		*tgt*	2	0.999277		*tilS*	1	0.99916
appB	2	0.999711		*aroH*	2	0.999092		*ybdR*	1	0.99903
ydcW	2	0.999231		*cysC*	2	0.999475		*nagE*	1	0.99931
yeeZ	2	0.999269		*lysA*	2	0.999450		*lolE*	1	0.99904
zipA	2	0.999522		*yheM*	2	0.999968		*ycgM*	1	0.99931
aspT	2	0.999148		*secF*	1	0.999374		*dhaL*	1	0.99925
secF	1	0.999127		*tesB*	1	0.999716		*exoX*	1	0.99922
nagB	1	0.999407		*seqA*	1	0.999245		*yedQ*	1	0.99908
nagE	1	0.999029		*artJ*	1	0.999745		*lysA*	1	0.99946
pepN	1	0.999764		*narX*	1	0.999231		*rpoD*	1	0.99918
hyaB	1	0.999856		*yciN*	1	0.999464		*cysG*	1	0.99930
hyaE	1	0.999478		*ydcW*	1	0.999126		*tag*	1	0.99998
hyaF	1	0.999504		*rspB*	1	0.999444		*rpmH*	1	0.99940
lolE	1	0.999685		*yodA*	1	0.999197		*asnC*	1	0.99945
phoP	1	0.999533		*yeeY*	1	0.999481		*cytR*	1	0.99969
dhaL	1	0.999690		*yeeZ*	1	0.999223				
rssA	1	0.999102		*yhjR*	1	0.999187				
oppF	1	0.999566		*ttk*	1	0.999612				
rspB	1	0.999647		*yieM*	1	0.999241				
ydgD	1	0.999316		*rplA*	1	0.999379				
rstA	1	0.999987		*creB*	1	0.999318				
sanA	1	0.999183								
cysP	1	0.999125								
yfiP	1	0.999219								
clpB	1	0.999009								
rpoD	1	0.999504								
yrbF	1	0.999078								
fdhE	1	0.999495								

[a]Genes that met the condition that the absolute correlation coefficient was 0.999 or higher, obtained by calculating the Pearson correlation coefficient between the gene

expression information and the image features extracted from the CNN model, are shown. The genes that correlated frequently with the image features are listed in descending order of number of appearances. The number of image features varied in the three folds as 948, 836, and 808, respectively.
